# Regulation of Agouti-Related Protein and Pro-Opiomelanocortin Gene Expression in the Avian Arcuate Nucleus

**DOI:** 10.3389/fendo.2017.00075

**Published:** 2017-04-13

**Authors:** Timothy Boswell, Ian C. Dunn

**Affiliations:** ^1^School of Biology, Newcastle University, Newcastle upon Tyne, UK; ^2^Royal (Dick) School of Veterinary Studies, Roslin Institute, University of Edinburgh, Easter Bush, UK

**Keywords:** melanocortin, AGRP, pro-opiomelanocortin, melanocortin 4 receptor, hypothalamus, birds, leptin

## Abstract

The arcuate nucleus is generally conserved across vertebrate taxa in its neuroanatomy and neuropeptide expression. Gene expression of agouti-related protein (AGRP), neuropeptide Y (NPY), pro-opiomelanocortin (POMC), and cocaine- and amphetamine-regulated transcript (CART) has been established in the arcuate nucleus of several bird species and co-localization demonstrated for AGRP and NPY. The proteins encoded by these genes exert comparable effects on food intake in birds after central administration to those seen in other vertebrates, with AGRP and NPY being orexigenic and CART and α-melanocyte-stimulating hormone anorexigenic. We have focused on the measurement of arcuate nucleus AGRP and POMC expression in several avian models in relation to the regulation of energy balance, incubation, stress, and growth. AGRP mRNA and POMC mRNA are, respectively, up- and downregulated after energy deprivation and restriction. This suggests that coordinated changes in the activity of AGRP and POMC neurons help to drive the homeostatic response to replace depleted energy stores in birds as in other vertebrates. While AGRP and POMC expression are generally positively and negatively correlated with food intake, respectively, we review here situations in some avian models in which AGRP gene expression is dissociated from the level of food intake and may have an influence on growth independent of changes in appetite. This suggests the possibility that the central melanocortin system exerts more pleiotropic functions in birds. While the neuroanatomical arrangement of AGRP and POMC neurons and the sensitivity of their activity to nutritional state appear generally conserved with other vertebrates, detailed knowledge is lacking of the key nutritional feedback signals acting on the avian arcuate nucleus and there appear to be significant differences between birds and mammals. In particular, recently identified avian leptin genes show differences between bird species in their tissue expression patterns and appear less closely linked in their expression to nutritional state. It is presently uncertain how the regulation of the central melanocortin system in birds is brought about in the situation of the apparently reduced importance of leptin and ghrelin compared to mammals.

## Introduction

Neural circuitry in the arcuate nucleus of the hypothalamus is well established in mammals as being particularly important for the regulation of energy balance (1, 2). Two neuronal cell types are involved. One synthesizes both agouti-related protein (AGRP) and neuropeptide Y (NPY), and the other produces α-melanocyte-stimulating hormone (α-MSH) and other peptides from the pro-opiomelanocortin (POMC) precursor together with cocaine- and amphetamine-regulated transcript (CART). AGRP and α-MSH peptides secreted from arcuate nucleus neurons bind to melanocortin receptors in the brain and collectively comprise the components of the central melanocortin system. AGRP/NPY neurons exert anabolic effects on food intake and body mass while POMC/CART neurons are catabolic. Evidence from non-mammalian vertebrate taxa suggests that the neuronal circuitry has been evolutionarily conserved both in its neuroanatomical location and in the genes the cells express. For example, AGRP, NPY, POMC, and CART mRNAs have all been localized in several teleost fish species in the lateral tuberal nucleus (NLT), the equivalent of the mammalian arcuate nucleus (3–6). Comparable observations have been made in birds, where the arcuate nucleus has historically been named the infundibular nucleus. Immunoreactive cell bodies and mRNA have been localized in the arcuate nucleus in several bird species for NPY, AGRP, and POMC (7–12). Furthermore, the co-expression of AGRP and NPY mRNA characteristic of laboratory rodents has been demonstrated in individual arcuate nucleus neurons in the Japanese quail (*Coturnix coturnix japonica*) (13). Less is known about CART in birds and its co-expression with POMC has yet to be formally demonstrated. However, immunoreactive CART neuronal cell bodies have been identified in the arcuate nucleus of the zebra finch (*Taeniopygia guttata*) (14).

We consider in this review the extent to which the evolutionary neuroanatomical conservation of the arcuate nucleus neurons implicated in energy balance in birds is conserved at the functional level, with an emphasis on our recent studies on the regulation of AGRP and POMC expression in several avian models.

## AGRP, POMC, and Energy Homeostasis

### Nutritional Sensitivity of AGRP and POMC Gene Expression

It is well established in laboratory rodents that neuropeptide gene expression in the arcuate nucleus AGRP/NPY and POMC/CART neurons is sensitive to nutritional state (physiologically reported in mammals by variation in plasma leptin concentrations) as part of a counter-regulatory response to loss of body energy stores during situations of negative energy balance such as fasting or food restriction (2). Comparable findings have been obtained in birds in response to both experimental food deprivation and chronic restriction. For example, AGRP mRNA was increased by food deprivation for 24 h in adult Japanese quail, a response also consistently observed after food deprivation for 24–48 h in domestic broiler chicks (11, 15–18). AGRP expression returned to baseline levels after 24 h refeeding (15–18). We have extended these observations to investigate the effects of chronic food restriction in growing broiler breeder hens. Food restriction of the parent hens of broiler chickens during the growth phase is a common practice in the poultry industry in order to mitigate poor reproductive performance when birds are fed *ad libitum* (19). We observed that AGRP mRNA was strongly increased in 12-week-old hens that had been maintained on an industrial food restriction regime for 6 weeks and gene expression returned to baseline after 2 days’ refeeding (20). A further comparison within the same experiment indicated that the level of AGRP mRNA was sensitive to feeding history: in two groups of hens sampled at the same body mass, AGRP gene expression was significantly higher in a group that had been maintained at an intermediate level of food restriction for 6 weeks compared to a group that had been maintained on commercial food restriction for 4 weeks before being released onto 2 weeks of *ad libitum* feeding. These results suggested that AGRP gene expression in broiler breeder hens is at least as sensitive to chronic food restriction as in mammals such as sheep, Siberian hamsters (*Phodopus sungorus*), rats, and golden spiny mice (*Acomys russatus*) (21–24). Furthermore, its expression is more closely linked to feeding state than reported in some rat studies of chronic food restriction where either no change was observed in AGRP mRNA, or where arcuate nucleus neuropeptide mRNAs had not returned to baseline levels after 4 weeks’ refeeding (25, 26).

It would be predicted from mammalian studies that food deprivation and restriction would exert an opposite, inhibitory, effect on POMC mRNA levels compared to AGRP mRNA because POMC gene expression induces a catabolic effect on energy balance (2). However, in birds, some studies have reported no change in POMC mRNA level after 24–48 h food deprivation in Japanese quail and broiler chicks (11, 17) while significantly decreased POMC expression has been observed in other broiler chick studies (15, 16, 18). For chronic food restriction, we detected no difference in POMC expression after 6 weeks’ restriction in our study of 12-week-old broiler breeder hens mentioned above, but another investigation reported a significant decrease after 7 days’ restriction in both 3-week-old broiler and layer chicks (20, 27). The greater variability in detecting altered POMC mRNA and the reduced magnitude of the change in POMC expression compared to that for AGRP in response to food deprivation or restriction may reflect a greater relative importance of increased AGRP expression in the counter-regulatory response to lost energy stores or, alternatively, a possibly greater role for regulatory control at the level of POMC-derived peptide secretion as has been reported in mammals (28).

### Nutritional Regulation of AGRP- and POMC-Derived Peptide Synthesis and Secretion

The significance of altered AGRP and POMC gene expression in response to manipulation of energy status is beginning to be understood in laboratory rodents. For example, the respective increased and decreased AGRP and POMC mRNA following a fast appears to be matched by parallel changes at the level of increased release of AGRP peptide and decreased release of α- and γ-MSH peptides (29–31) and by, respectively, increased and decreased firing of AGRP and POMC neurons (32, 33). Furthermore, optogenetic approaches have demonstrated that feeding behavior can be directly induced or inhibited by light activation of AGRP and POMC neurons, respectively (34). In contrast, most studies of the central melanocortin system in birds have focused on mRNA measurements as an indicator of the activity of the neurons. However, a few investigations have demonstrated the presence of central melanocortin system peptides in the avian arcuate nucleus. For example, AGRP immunoreactivity was identified in the Khaki Campbell duck (*Anas platyrhynchos*) arcuate nucleus (12), and evidence for altered synthesis of AGRP peptide is available for the ring dove (*Streptopelia risoria*), where increased numbers of AGRP-immunoreactive cell bodies were observed in the medio-basal hypothalamus following a 48-h fast and also during the post-hatching phase of the reproductive cycle when the parent birds are in negative energy balance as a result of the demands of feeding offspring (10, 35). Relatively little is known in birds about the processing of POMC-derived peptides in the hypothalamus and their relative importance for energy balance regulation. However, immunoreactive neuronal cell bodies for α-MSH, β-endorphin, and N-terminal POMC (pro-γ-MSH) were detected in the arcuate nucleus of broiler chickens (7), and co-localization of POMC mRNA and α-MSH peptide was observed in individual arcuate nucleus neurons in Japanese quail (11).

Given the general lack of information in birds about AGRP and POMC signaling above the mRNA level, the significance of changes in gene expression in relation to energy status is generally inferred from the mammalian literature and from the behavioral effects of the encoded peptides. There is behavioral evidence that domestic pigeons and Japanese quail eat more in a refeeding period after food deprivation compared to control birds that had been allowed to feed freely over the experimental fasting phase (13, 36). This increased food intake combined with knowledge about the effects of energy deprivation on AGRP and POMC gene expression is consistent with the idea that fasting stimulates food intake by increased secretion of AGRP peptide and, in some situations, reduced secretion of POMC-derived peptides such as α-MSH.

### Central Melanocortin Receptors

The avian central melanocortin system peptides appear to exert their effects by acting on melanocortin receptors in the hypothalamus as in mammals. Characterization of the pharmacological properties of the five chicken melanocortin receptor subtypes *in vitro* revealed a relatively greater affinity for ACTH-derived peptides than for α-MSH compared to their mammalian orthologs (37). This might suggest a more significant role in birds for ACTH as a central melanocortin receptor ligand. Studies have not been performed to localize or quantify ACTH peptide in the avian hypothalamus, but its synthesis and secretion have been reported in the hypothalamus of laboratory rats (28), and an inhibitory effect on feeding of the centrally administered peptide has been observed in rats, domestic pigeons (*Columba livia*), and broiler chicks (38–40). Another POMC peptide, β-endorphin, has been localized in the chicken arcuate nucleus, as noted above (7). It stimulates food intake after central injection of the ostrich and mammalian peptide, respectively, in the domestic pigeon and the chicken (41, 42). Further investigation is needed to explore the context and significance of β-endorphin secretion from POMC/CART neurons.

Pharmacological studies have been performed in birds on the melanocortin 4 receptor (MC4R), the melanocortin receptor most strongly associated with the regulation of food intake in mammals. The synthetic compounds HS014 and HS024 were identified *in vitro* as selective antagonists and melanotan II (MTII) as a potent agonist in the chicken (37). The predicted stimulatory effects on food intake for the antagonist, and inhibitory effects for the agonist, have been confirmed for HS014 and MTII in ring doves and in broiler and layer chickens following central or peripheral injection (27, 35, 43). Several studies have investigated the effect of central injection of MSH peptides on food intake in domestic chicks. Inhibitory effects of α-, β-, and γ2-MSH have been observed (44–47) with reports of differential sensitivity between broiler and layer and high and low-growth lines (48, 49). However, while the amino acid sequence of α-MSH is conserved across vertebrates, the chicken sequences for β- and γ2-MSH differ between birds and mammals and appear to have a less potent effect on food intake in chicks when administered centrally compared to mammalian versions (43). This suggests a more prominent role for α-MSH among the avian POMC-derived peptides. As in mammals, there is evidence for competition between the α-MSH agonist and the AGRP antagonist at central melanocortin receptors because central injection of AGRP dose-dependently attenuated the inhibitory effect on feeding of α-MSH in domestic chicks (45). Central injections of AGRP alone stimulated food intake, as expected, in the ring dove but with an apparently lower potency than observed in laboratory rodents, which may reflect the use of heterologous human AGRP fragments in the ring dove experiment (35). Layer chicks were observed to be more sensitive in their feeding response to central AGRP injection than broiler chicks (45).

While pharmacological and behavioral studies suggest that the actions of AGRP- and POMC-derived peptides, particularly α-MSH, are conserved between birds and mammals, knowledge is lacking in birds about the site of action of the peptides and the central melanocortin receptor subtypes that naturally mediate their signaling effects: the projections of AGRP/NPY and POMC/CART neurons have not been studied, and the central melanocortin receptors involved have not been precisely localized. Both MC4R and melanocortin 3 receptor (MC3R) mRNA have been quantified in the chicken hypothalamus in real-time PCR studies (15, 17, 50, 51) and the melanocortin 5 receptor more generally in the brain (50, 52). Increased MC4R gene expression has been observed after 48 h food deprivation in broiler chicks (15, 17), which may be in response to decreased agonistic drive. Little is known about the possible role of the MC3R in the regulation of energy balance in birds, but its hypothalamic expression was higher in chickens lines selected for low compared to high body weight (50, 51).

## Regulation of AGRP and POMC Expression by Incubation, Stress, and Preparation for Migration

### Effects of Incubation and Stress

While the dynamic coordinated changes in arcuate nucleus AGRP and POMC gene expression in response to energy shortage are well established in mammals and conserved in birds, less is known about the occurrence and influence of altered basal expression of these genes. Uniquely avian models are available to test the hypothesis that variation in the levels of AGRP and POMC mRNA measured in individuals with free access to food promotes the expression of natural seasonal changes in feeding behavior and metabolism. We have recently investigated this in a chicken strain that exhibits natural incubation behavior. Hens incubate their eggs over a 3-week period that is associated with a suite of behavioral and physiological changes known as “broodiness” (53). Hens spend an increasing amount of time sitting on their eggs and cease laying. Linked to this is the expression of natural anorexia when birds reduce their food intake and reduce body mass over the incubation period (54). Experimental food deprivation and refeeding in junglefowl hens suggested that the level around which body mass is defended (or set point) is reduced during incubation, and the concept was extended to other vertebrate species of natural “animal anorexias” that are expressed in phases of life history such as hibernation or territorial defense where the time available to feed is limited (55, 56).

We drew on these studies to investigate how AGRP and POMC gene expression changes during the incubation phase in hens (57). One possible outcome was that there would be no change in gene expression because in birds with free access to food, body mass is at its appropriate defended level despite the fact that body mass is lower: altered AGRP and POMC expression would only be expected in response to perturbation such as food deprivation or restriction. Alternatively, it was possible that changes in basal gene expression play a role in promoting the loss of body mass. In this case, increased POMC expression would be expected, combined with unaltered, or reduced, AGRP expression. We controlled for possible confounding effects of, respectively, enlarged and regressed ovaries in laying and incubating hens by pair-feeding two groups of laying hens to the amount of food eaten voluntarily by incubating birds (57). This resulted in ovarian regression in those groups that matched that shown in the incubating hens. One of the pair-fed groups was allowed to refeed for 5 days so that food intake and body mass stabilized to reveal the natural *ad libitum* food intake level in birds with regressed ovaries. In birds sampled 21 days after the onset of incubation, POMC gene expression was increased to a level on the border of statistical significance in the incubating hens compared to the two control groups, consistent with the idea that this is linked to an increased anorexic drive. However, unexpectedly, AGRP mRNA was higher in both incubating and pair-fed birds, compared to the re-fed control group. This finding is interesting in suggesting that increased AGRP gene expression (and the assumed associated increase in AGRP peptide signaling) does not necessarily result in increased food intake. This situation was not unique to incubation. A related experiment arose from our observation that hens transferred from single housing in a cage to housing in a pen showed reduced food intake in their new environment presumably because they perceived the transfer as stressful (57). Measurements of AGRP and POMC gene expression 6 days after the housing transfer showed results comparable to the incubation experiment, this time with significantly increased POMC mRNA combined with increased AGRP expression. This result is again consistent with increased POMC expression contributing to anorexic drive that leads to the reduced food intake. The fact that AGRP gene expression is increased during incubation and after housing transfer suggests that its sensitivity to reduced energy availability is maintained despite an apparent change in the defended set point for body mass during incubation. The normal stimulatory effects of AGRP expression on food intake may be overridden by a relatively greater inhibitory influence of the increased POMC expression and, in the case of incubation, a possible inhibitory influence on feeding of hypothalamic vasoactive intestinal polypeptide, the expression of which is causatively linked to incubation behavior in birds (53). The fact that AGRP gene expression is increased in these situations may be of adaptive significance in promoting more rapid restoration of energy stores when incubation ends and as part of the recovery from the effects of a stressor. For incubation, it is also possible that increased AGRP mRNA is linked to altered daily patterns of behavior during incubation when expression of ingestive behavior is confined to two daily recesses from nest sitting but during which feeding may be relatively intense (53).

### Photoperiodic Effects and Migratory Physiology

Another opportunity provided by avian models to investigate the possibility of seasonal regulation of AGRP and POMC gene expression is represented by species that show increased appetite and fat deposition as preparation for migratory flight (58). Laboratory studies in captive birds have revealed that the increased appetite (hyperphagia) and fat deposition that occur before migration are stimulated by changes in daylength and appear to involve changes in the level around which body mass is regulated as has been suggested for incubation (59). Seasonal changes in reproductive physiology mediated by photoperiod in birds and seasonal mammals have been linked to release of thyroid-stimulating hormone from the pars tuberalis of the pituitary gland. This results in conversion within the medio-basal hypothalamus of thyroxine into triiodothyronine (T3) that promotes release of gonadotropin-releasing hormone from neuron terminals in the median eminence (60). It is possible that locally increased tissue concentrations of T3 mediate seasonal cycles in appetite and fat deposition in addition to reproduction. This is suggested by a mammalian study of Siberian hamsters that received hypothalamic implants of T3. This procedure on short day animals induced changes in body mass characteristic of exposure to long photoperiods (61). Furthermore, there is evidence in domestic chicks that experimentally increased T3 stimulates hypothalamic AGRP gene expression both *in vivo* and *in vitro* (62). However, there is limited evidence in hamsters for an important role for the arcuate nucleus and its neuropeptides in driving seasonal cycles in food intake and body mass (63). We are currently investigating whether the situation is similar in birds by quantifying AGRP gene expression in Gambel’s white-crowned sparrows (*Zonotrichia leucophrys gambelii*) after photostimulation. This will test whether increased AGRP gene expression is associated with seasonally increased food intake in this migratory species.

## Influence of Growth and Sex on AGRP and POMC Expression

**Figure 1:**
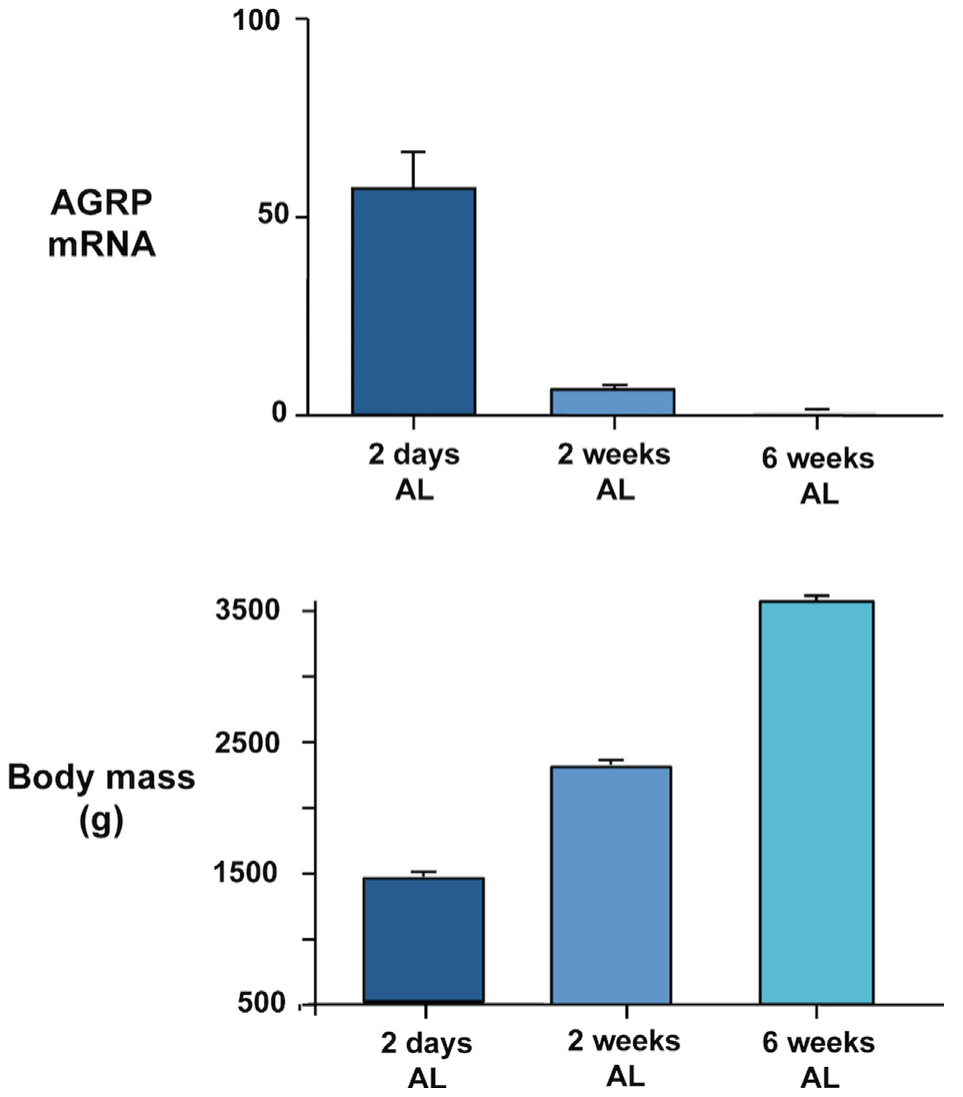
**Inverse relationship between AGRP gene expression quantified in dissected basal hypothalamus by real-time PCR (upper panel) and body mass (lower panel) in female broiler breeder chickens (Ross 308 line)**. Birds were maintained on a commercial food restriction program from hatch before being transferred to *ad libitum* (AL) feeding at 2 days, 2 weeks, and 6 weeks before the birds were killed at 12 weeks of age. Further experimental details are provided in Ref. (20).

In addition to a possible role of basal changes in gene expression in regulating reproductive and seasonal changes in food intake and body weight, our recent studies suggest that AGRP and POMC expression may be related to growth. In our chronic food restriction experiment on broiler breeder hens (20) reviewed in relation to energy homeostasis above, we noted that AGRP gene expression was inversely related to body mass in the groups maintained on *ad libitum* feeding at the time of sampling (Figure 1). Birds were the same age at the time they were killed and had all been maintained on commercial restricted feeding for the first 6 weeks. The highest body mass was attained in the birds fed *ad libitum* over the next 6 weeks and the lowest in birds that were only allowed to refeed for 2 days. AGRP mRNA remained significantly elevated in birds fed *ad libitum* for 2 weeks compared to 6 weeks. Thus, the level of AGRP expression could be regarded as a measure of the growth potential of the different experimental groups, with the highest expression in birds that were furthest away from the natural growth trajectory. We have further evidence for this at the genetic level. When we compared AGRP gene expression between males and females in 12-week-old broiler breeder chickens that had been re-fed for 2 days after food restriction, expression was significantly higher in males and this difference was replicated in *ad libitum*-fed fully mature chickens of another genetic strain (64). The higher AGRP expression in males is consistent with the fact that they grow faster and attain a higher mature body mass. It would therefore be predicted that AGRP mRNA would be higher in fully fed birds in chicken strains that grow more rapidly. We obtained evidence for this from trait linkage analysis of birds from a broiler-layer cross that differed in growth rate (65). We identified a genotype that explained 19% of the difference in body mass between the lines that was associated with lower global tissue expression of the cholecystokinin A receptor (CCKAR) in the high-growth haplotype. We demonstrated that the high-growth birds were less sensitive to the inhibitory effects on food intake of intraperitoneal cholecystokinin (CCK) injection and that they showed significantly higher expression of AGRP (but no difference in POMC mRNA). This suggests that the tone of CCK signaling influences AGRP expression. It could be predicted that increased AGRP expression is causative in generating higher growth by stimulating food intake. However, we have been unable to find a consistent difference in daily food intake between the lines. This demonstrates again, as we observed for incubation, that high AGRP gene expression can be dissociated from increased feeding. Any effects of AGRP on growth rate must therefore be independent of food intake. The mechanisms involved are currently unclear and point to a need in birds to investigate the effects of central melanocortin system signaling on metabolic aspects of energy balance regulation distinct from feeding behavior.

While our observations linking AGRP expression to growth in birds are preliminary and require further investigation, they serve to highlight a possible involvement of the central melanocortin system in growth regulation that has received limited attention in vertebrates compared to its more general effects on energy homeostasis. Experimental inactivation or natural mutation of the MC4R in laboratory mice and humans is associated with increased linear growth (66, 67). The mechanisms underlying this effect are not fully understood but appear to be independent of growth hormone secretion and involve hyperinsulinemia (68, 69). More direct evidence for a link between the central melanocortin system and growth has been obtained from teleost fish for which it has been reported that transgenic overexpression of the natural melanocortin receptor antagonist agouti-signaling protein reversed the pattern of sexually dimorphic growth in zebrafish (*Danio rerio*) (70) and that AGRP and POMC neurons project directly to the pituitary gland (71). Transgenic overexpression of AGRP itself in zebrafish led to both obesity and increased linear growth, while suppression of AGRP expression reduced larval growth rate and was mediated through the MC4R (71, 72). Thus it seems that AGRP exerts more pleiotropic effects on hormonal axes through the pituitary gland in teleosts compared to mammals and more investigation is needed to determine whether this applies to non-mammalian vertebrates more generally including birds.

## Regulation of AGRP and POMC Expression by Metabolic Hormones, Hypothalamic Energy Sensing, and Gut Fill

### Leptin

Less is known in birds than mammals about the regulatory feedback signals to the arcuate nucleus that mediate the physiological changes in AGRP and POMC gene expression reviewed above. This is due in part to uncertainty over the status of leptin in birds, with leptin being a key regulator of the central melanocortin system in mammals (2). Leptin genes have recently been identified in several avian species after a 20-year search (73–77). However, they have low (about 30%) amino acid sequence identity with mammalian leptins, with variable tissue expression patterns between species and limited expression in adipose tissue (78). Mammalian leptins inhibit food intake in birds after central or peripheral injection but peripheral infusion of “chicken leptin” (later shown to be mouse leptin) had no effect on AGRP and POMC gene expression in young broilers (79). Thus, unlike the situation in mammals, there is currently no direct evidence to support an action of leptin on AGRP and POMC gene expression, but further investigation is needed that takes into account the new information on avian leptin.

### Ghrelin

A pronounced difference between birds and mammals is also apparent for the effects of ghrelin on food intake. There is commonality in that, as in mammals, ghrelin is expressed in the avian stomach (proventriculus) and its gene expression and circulating protein are increased by fasting and decreased by refeeding in layer chickens and Japanese quail (80). Also, in free-living garden warblers (*Sylvia borin*) sampled at a stopover site during spring migration, plasma ghrelin concentrations were positively associated with higher fat scores (81). However, unlike the situation in mammals, central and peripheral injections of mammalian and chicken ghrelin decrease, rather than stimulate, feeding (80, 81). There is evidence in mammals for a regulatory influence of ghrelin on AGRP/NPY neurons (82). Expression of the ghrelin receptor (GHSR) has been detected in the domestic chick hypothalamus (83), but its localization there, including in the arcuate nucleus, is unknown. The inhibitory effect of centrally administered ghrelin on food intake appeared to be mediated *via* corticotropin-releasing factor rather than through AGRP/NPY neurons because ghrelin injection did not influence NPY gene expression (84).

### Hypothalamic Energy Sensing

At the local tissue level, there is suggestive evidence for a link in birds between arcuate nucleus neuropeptide gene expression and hypothalamic energy sensing as there is in mammals. Immunoreactivity for the energy sensor AMP-activated protein kinase (AMPK) was observed in the chicken arcuate nucleus and food deprivation for 48 h increased phosphorylated AMPKα in parallel with AGRP mRNA (17, 85). In broiler chicks, 24 h food deprivation led to increased expression of the AMPK subunit mRNAs AMPKα2, AMPKβ1, AMPKβ2, and AMPKγ1 along with, respectively, increased and decreased AGRP and POMC mRNAs (16). When domestic broiler chicks were fed with the AMPK inhibitor α-lipoic acid (α-LPA), hypothalamic AMPKα1 mRNA was decreased along with food intake, confirming α-LPA’s inhibitory effect (86). However, the pattern of expression of AGRP and POMC was opposite to that predicted for a regulatory effect of AMPK. The same study (86) also confirmed the expression in the chick hypothalamus of hypoxia-inducible factor-1α, a nuclear transcription factor that influences POMC gene expression in mammals in response to local hypoxia (87). More generally, microarray and real-time PCR analysis of gene expression in the hypothalamus of broiler chicks food-deprived for 48 h suggested that fasting induces metabolic switching (16, 18). Genes associated with fatty acid oxidation and inhibition of glycolysis were upregulated, and those linked to fatty acid synthesis and transport downregulated, in parallel with increased AGRP and reduced POMC gene expression. The suggestion of metabolic switching was supported by central injection of compounds influencing glycolysis and fatty acid oxidation (18). Injection of α-LPA decreased food intake, which is consistent with the finding of the dietary administration study above and with α-LPA stimulating glycolysis through inhibition of pyruvate dehydrogenase kinase isoform 4 (18). In contrast, injection of the glycolytic inhibitor 2-deoxyglucose stimulated food intake. The possible importance of the metabolic sensor sirtuin 1 in mediating the metabolic switching was suggested by the fact that administration of its inhibitor NADH and activator NAD+, respectively, decreased and increased food intake (18). However, the effect of these manipulations on neuropeptide gene expression was not reported so that it is not clear to what extent the changes in food intake observed are directly attributable to regulatory effects on AGRP/NPY and POMC/CART neurons. Thus, overall, although suggestive, evidence for a direct effect of metabolic sensing on AGRP and POMC gene expression is lacking and further investigation is needed.

### Local Hypothalamic Signaling

Signals that may influence central melanocortin system gene expression within the hypothalamus have been explored by microarray and pathway analysis in newly hatched broiler chicks that were food deprived for up to 48 h and re-fed (15). Functional interactions, supported by hypothalamic cell culture experiments, were identified within a network of six genes encoding the neuropeptide relaxin-3, the neuropeptide receptors NPY5R and somatostatin receptor 5, and the β2 adrenergic and metabotropic glutamate receptor 8 neurotransmitter receptors together with POMC, which appeared to play a central role. POMC expression was downregulated by fasting while the other genes were upregulated.

### Insulin

For metabolic hormones, evidence is needed for co-expression of hormone receptors in individual AGRP/NPY and POMC/CART neurons. So far, this has been established only for insulin. Before the discovery of leptin, insulin was favored in mammals as a long-term regulator of energy balance because fasting, or basal, concentrations report body fat content, the hormone is transported into the brain, and insulin receptors are present on AGRP/NPY neurons (2). It is less clear whether it plays a similar role in the long-term regulation of energy balance of birds: although circulating insulin concentrations are correlated with food intake in chickens, they did not differ under fasting conditions between selected lines of fat and lean birds (88). However, central injection of insulin decreased food intake in layer chicks and the effect was blocked by coadministration of the MC4R antagonist HS014 (89, 90). A direct effect of insulin on arcuate nucleus neurons was suggested by the demonstration of co-localization of insulin receptor immunoreactivity with that of NPY, and with α-MSH in individual neurons (91). Insulin stimulates POMC gene expression and inhibits that of AGRP and NPY in the brain of laboratory rodents (92), and this regulatory influence appears to have been conserved to some extent in birds. Thus, in the chick studies, central insulin injections consistently stimulated POMC expression, but a decrease in NPY mRNA was observed in one study and not another, and no decrease in AGRP mRNA was detected (89, 90). Overall, however, the results suggest an involvement of insulin in the response of POMC/CART and possibly AGRP/NPY neurons to fasting.

### Corticosterone and Thyroid Hormones

Other metabolic hormones that have a regulatory influence on AGRP/NPY and POMC/CART neurons in mammals are corticosterone and thyroid hormones. Circulating corticosterone is increased by fasting in birds and mammals and, respectively, increases and decreases AGRP and POMC gene expression in laboratory rodents (93, 94). There is evidence in domestic chicks for a suppressive effect of corticosterone on POMC expression (62, 95). However, there is more variability in the response of AGRP gene expression to centrally or peripherally administered corticosterone or to the glucocorticoid receptor agonist dexamethasone (62, 95–97). Thyroid hormones have already been mentioned in the context of preparation for migration above. There is evidence in both birds and mammals for a stimulatory effect of T3 on AGRP gene expression (63, 98). Thus, overall, there is some commonality in the regulatory influences of corticosterone and T3 on AGRP/NPY and POMC/CART neurons between birds and mammals.

### Cholecystokinin

We identified a possible link between signaling by the gut peptide CCK and AGRP expression in the context of growth as reviewed above (65). It is unclear whether the increased AGRP expression we observed in high-growth haplotype chickens is a secondary consequence of reduced CCKAR expression and experimental manipulations of CCK signaling in other chicken strains are needed. In mammals, there is evidence for an involvement of the central melanocortin system, both in the hindbrain and the hypothalamus, in the inhibitory effects of CCK on feeding because MC4R knockout mice show a reduced sensitivity (99, 100).

### Gut Fullness Effects

In addition to feedback effects by metabolic hormones, we have recently investigated the possibility for a sensitivity of AGRP gene expression to signals arising from gut fullness. This has been in the applied context of attempting to improve the welfare of broiler breeder chickens that experience prolonged hunger during the industrial practice of food restriction when birds receive a limited ration of food per day. As an alternative, it is possible to use alternative diets containing a high proportion of food that is normally high in fiber and of low energy density (101). This could potentially mitigate some of the undesirable effects of constant hunger by providing more total food and therefore more opportunity for the expression of natural foraging and ingestive behaviors. However, it is uncertain whether birds fed on such diets still experience a “metabolic hunger.” To address this, we provided restricted-fed 12-week-old broiler breeder birds with *ad libitum* access to food for 2 days compared to birds re-fed a diet over the same time period that was diluted with the non-nutritive bulking agent ispaghula husk, and to birds that remained on a restricted diet (64). We measured significantly increased AGRP expression and decreased POMC expression compared to fully fed controls in both food-restricted birds and in those receiving the dietary bulking agent. This suggests that AGRP and POMC gene expression is insensitive to mechanosensory signals relating to gut fullness. We are performing further experiments to confirm this using other more industrially relevant diet formulations.

## Conclusion

The striking neuroanatomical conservation among vertebrates of the arcuate nucleus AGRP/NPY and POMC/CART neurons appears to be accompanied in birds by functional conservation in these cells of a coordinated signaling response to energy deprivation whereby AGRP/NPY neurons are stimulated and POMC/CART neurons are inhibited, which promotes replacement of lost energy stores when food becomes available. However, the picture is somewhat incomplete in birds compared to mammals with investigations tending to be skewed toward the measurement of gene expression rather than being focused on secretion of the peptides. Information is lacking on the connections of the neurons, both within and outside the hypothalamus, and little is known about the metabolic and energetic effects of the arcuate nucleus peptides distinct from their effects on food intake. Knowledge in birds has also been limited by a lack of availability of transgenic methods to assess the effects on energy balance of experimental genetic activation and inactivation, although such methods now appear to be close to routine application (102).

The use of unique avian models to explore aspects of the regulation of the central melanocortin system has highlighted possible differences from mammals and emphasized adaptations that may be representative of those in other non-mammalian vertebrates. These include changes in basal expression of AGRP and POMC in relation to seasonal changes in food intake, energy balance, and reproduction that tend not to be apparent in mammalian models (63). There are also situations in birds in which AGRP gene expression is dissociated from the pattern of food intake, particularly in relation to sexually dimorphic growth, which widens the perspective of investigations into the functions of the melanocortin system in birds and other vertebrates beyond the regulation of appetite.

The apparent functional conservation of changes in central melanocortin system gene expression in response to energy shortage is somewhat puzzling from the evidence available on how the system is regulated by feedback signals. Circulating leptin is acknowledged to play a prominent regulatory role on the system in mammals whereas in birds its sites of synthesis are variable and less focused on adipose tissue, and it appears to act more as an autocrine/paracrine signaling factor than as a circulating hormone (78). The recent discovery of avian leptin genes together with the knowledge that the pattern of expression is more representative of other non-mammals than mammals offers the opportunity for the regulation of the avian melanocortin system to be viewed from a new perspective. More commonality between birds and mammals is evident in the regulatory effects of other metabolic hormones such as insulin, corticosterone, and T3. However, it is presently unclear whether these hormones exert the main regulatory effects on the system with diminished input from leptin and ghrelin, or whether other regulatory mechanisms are present that are either ancestral and representative of other non-mammalian vertebrates rather than mammals or are unique and linked to the general adaptations that have evolved in birds to support flight.

## Author Contributions

TB wrote the article with substantial intellectual and editorial input from ID on its content and structure. Both authors approved the work for publication.

## Conflict of Interest Statement

The authors declare that the research was conducted in the absence of any commercial or financial relationships that could be construed as a potential conflict of interest.
